# Nanoparticulate matter exposure results in white matter damage and an inflammatory microglial response in an experimental murine model

**DOI:** 10.1371/journal.pone.0253766

**Published:** 2021-07-02

**Authors:** Michelle Connor, Krista Lamorie-Foote, Qinghai Liu, Kristina Shkirkova, Hans Baertsch, Constantinos Sioutas, Todd E. Morgan, Caleb E. Finch, William J. Mack

**Affiliations:** 1 Department of Neurosurgery, Washington University School of Medicine, St. Louis, Missouri, United States of America; 2 Zilkha Neurogenetic Institute, University of Southern California, Los Angeles, California, United States of America; 3 Department of Civil and Environmental Engineering, Viterbi School of Engineering, University of Southern California, Los Angeles, California, United States of America; 4 Leonard Davis School of Gerontology, University of Southern California, Los Angeles, California, United States of America; 5 Department of Neurological Surgery, Keck School of Medicine, University of Southern California, Los Angeles, California, United States of America; Instituto Cajal-CSIC, SPAIN

## Abstract

Exposure to ambient air pollution has been associated with white matter damage and neurocognitive decline. However, the mechanisms of this injury are not well understood and remain largely uncharacterized in experimental models. Prior studies have shown that exposure to particulate matter (PM), a sub-fraction of air pollution, results in neuroinflammation, specifically the upregulation of inflammatory microglia. This study examines white matter and axonal injury, and characterizes microglial reactivity in the corpus callosum of mice exposed to 10 weeks (150 hours) of PM. Nanoscale particulate matter (nPM, aerodynamic diameter ≤200 nm) consisting primarily of traffic-related emissions was collected from an urban area in Los Angeles. Male C57BL/6J mice were exposed to either re-aerosolized nPM or filtered air for 5 hours/day, 3 days/week, for 10 weeks (150 hours; n = 18/group). Microglia were characterized by immunohistochemical double staining of ionized calcium-binding protein-1 (Iba-1) with inducible nitric oxide synthase (iNOS) to identify pro-inflammatory cells, and Iba-1 with arginase-1 (Arg) to identify anti-inflammatory/ homeostatic cells. Myelin injury was assessed by degraded myelin basic protein (dMBP). Oligodendrocyte cell counts were evaluated by oligodendrocyte transcription factor 2 (Olig2). Axonal injury was assessed by axonal neurofilament marker SMI-312. iNOS-expressing microglia were significantly increased in the corpus callosum of mice exposed to nPM when compared to those exposed to filtered air (2.2 fold increase; p<0.05). This was accompanied by an increase in dMBP (1.4 fold increase; p<0.05) immunofluorescent density, a decrease in oligodendrocyte cell counts (1.16 fold decrease; p<0.05), and a decrease in neurofilament SMI-312 (1.13 fold decrease; p<0.05) immunofluorescent density. Exposure to nPM results in increased inflammatory microglia, white matter injury, and axonal degradation in the corpus callosum of adult male mice. iNOS-expressing microglia release cytokines and reactive oxygen/ nitrogen species which may further contribute to the white matter damage observed in this model.

## Introduction

Exposure to ambient air pollution has deleterious effects on multiple organ systems and is estimated to lead to 3.3 million premature deaths per year [1]. Human studies have demonstrated that particulate matter exposure is associated with neurodevelopmental and neurodegenerative diseases, as well as decreased cognitive function [2–12]. Air pollution has been shown to result in neuroinflammation and oxidative stress in humans and animal models [3, 13–17]. White matter may be particularly vulnerable to this neurotoxicity [3, 14, 18–21].

Microglia are the resident immune cells of the central nervous system and continuously surveil the brain microenvironment [22, 23]. Microglia adopt various states of reactivity in response to a number of stimuli, including ischemia, neuronal death, hemorrhage, toxins, infections, and particulate matter [13, 14, 24, 25]. In response to environmental stimuli, microglia undergo a rapid change in morphology, gene expression, and function that supports both injury and repair [25, 26]. Microglia can adopt a wide variety of phenotypes, ranging from pro-inflammatory to anti-inflammatory and neuroprotective [26]. Microglia adopt a pro-inflammatory phenotype in response to lipopolysaccharide (LPS), interferon gamma (IFN-γ), and other inflammatory stimuli. This polarization results in release of cytokines and reactive oxygen and nitrogen species, leading to neuronal injury, white matter damage, and further microglial activation [26, 27]. Pro-inflammatory microglia are characterized by an upregulation of inducible nitric oxide synthase (iNOS), tumor necrosis factor (TNF), and interleukin-6 (IL-6) [28]. Microglia adopt an anti-inflammatory phenotype in response to IL-4, IL-10, and transforming growth factor-β (TGF-β). Anti-inflammatory microglia are characterized by the upregulation of arginase-1 (Arg), CD206, and IL-10. This subtype is typically considered to be neuroprotective and promotes remyelination, and cellular and vascular regeneration after injury [28–31]. Pro-inflammatory and anti-inflammatory microglial markers, such as iNOS and arginase-1, are not specific to microglia and are expressed by multiple cell types in the central nervous system [32–34].

Ionized calcium-binding protein-1 (Iba-1) is an actin-cross linking protein that is expressed in all microglia and upregulated in reactive microglia. Iba-1 functions in membrane ruffing, which is essential for microglia to undergo morphological changes in response to injury [35, 36]. Iba-1 is utilized as a marker for both surveilling and reactive microglia [36]. Double immunofluorescent staining of Iba-1 with iNOS and arginase-1 markers can be performed to identify pro-inflammatory and anti-inflammatory microglia [37].

Our previous murine studies have demonstrated that 150 hours of traffic- derived nPM exposure results in a specific microglial response (in the absence of an astrocyte response) in the corpus callosum of mice. This is evidenced by an increased number of Iba-1 positive cells, as well as morphological changes that suggest microglial reactivity [14]. In the present study, we aim to assess white matter and axonal injury, as well as characterize the microglial response in the corpus callosum of mice exposed to nPM for 150 hours. We hypothesize that nPM exposure results in white matter damage and axonal degradation. We propose that the pro-inflammatory phenotype is dominant in reactive microglia due to the ongoing neurotoxic insult of nPM exposure.

## Materials and methods

All mice were randomized to either filtered air or nPM exposure groups. All animal caretakers and investigators were blinded to the exposure allocation.

### Animals

Male C57BL/6J mice aged 10 weeks were used in this study. Experiments were performed in accordance with the University of Southern California Institutional Care and Use Committee (IACUC) and the Guide for the Care and Use of Laboratory Animals (NIH). The protocol was approved by the University of Southern California IACUC (Protocol Number: 20235). Mice were purchased from Jackson Laboratories and housed with the University of Southern California Department of Animal Resources. No previous procedures were performed on the mice. Mice were group housed with four mice in each cage and kept on a 12-hour light-dark cycle. Mice were randomized to either nPM (n = 18) or filtered air (n = 18) exposure cohorts. The study’s primary endpoint was white matter injury and the secondary endpoints were axonal degradation and microglial activation. Sample size was calculated to determine a difference between exposure cohorts according to the primary endpoint, white matter injury.

Preliminary data on white matter injury in pilot nPM studies (part of a larger study examining the effects of nPM and chronic cerebral hypoperfusion, manuscript under review) was used to perform a power analysis. We estimated that nPM exposure effects would be similar to the injury seen in our pilot data. According to power analysis, each arm of the study (nPM/filtered air) would require 15 animals to demonstrate the expected mean group difference (two-sided alpha = 0.05, 80% power). To account for variation in sensitivity analysis, 18 mice were selected per group. In the experimental paradigm, mice were exposed to either nPM or filtered air for 5 hours per day, 3 days per week, for 10 weeks (150 cumulative hours). Animals had free access to food and water, except during nPM/filtered air exposure periods.

Within 72 hours of the last exposure, mice were humanely euthanized with an intraperitoneal injection of ketamine and xylazine. Mice were transcardially perfused with PBS+heparin saline solution followed by fixative solution (4% paraformaldehyde in 0.01 mol/L PBS buffer). Brains were dissected, stored in paraformaldehyde for 24 hours at 4°C, dehydrated in 70% ethanol, and sent to pathology for paraffin embedding. Mice that died during the exposure period were excluded from analysis based on prospectively established criteria. No mice died or were euthanized during the exposure period. Humane euthanasia was to be given if any animal appeared to be in distress during the exposure period per protocol guidelines. Mice were monitored daily. No adverse events occurred. All mice were included in analysis and there were no outliers.

### Nanoparticulate matter collection

nPM, a subfraction of ambient air pollution measuring <200nm in diameter, was collected in an urban area in Los Angeles primarily impacted by traffic-related emissions [38]. A high-volume ultrafine particle sampler collected particles at 400L/min on pretreated Teflon filters (8x10”, PTFE, 2μm pore) using a multiple rectangular (slit) geometry jet conventional impactor to remove particles larger than 200nm [39]. Filters were soaked in Milli-Q deionized water (resistivity 18.2MW, total organic compounds <10ppb, particle free, endotoxin levels <1units/mL, endotoxin-free glass vials) for 30 minutes to transfer nPM into aqueous suspension, and then resuspended through vortexing (5 minutes) and sonication (30 minutes). The suspension was stored as pooled, frozen stock at -20°C for over 3 months, according to recommended US Environmental Protection Agency procedures. *Limulus* amoebocyte assay confirmed no endotoxin was detected in the suspensions (LPS <0.02EU/mL). The presence of endotoxin in nPM suspensions may expose mice to a potent inflammatory agent during nPM exposure. This may subsequently skew results and result in a false inflammatory response in mice. Sterile filters were sham extracted and stored as a control.

### Nanoparticulate matter exposure

Mice were exposed to either re-aerosolized nPM or filtered air in sealed, temperature controlled, whole-body exposure chambers. nPM was re-aerosolized using a HOPE nebulizer (B&B Medical Technologies, Carlsbad, CA) with compressed particle-free filtered air. 15 L/min of aerosol flow was generated, with 10 L/min drawn through the animal exposure chamber, and the remaining 5 L/min diverted for continuous monitoring of particle size and concentration. A scanning mobility particle sizer (SMPS model 3080, TSI Inc., Shoreview, MN) was used to monitor and maintain an average nPM mass concentration at approximately twice that of a busy roadway (330 ± 25 μg/m^3^). nPM was concurrently collected on Teflon and quartz filters, and numerous assays were used to characterize composition of re-aerosolized nPM. Pre- and post- weighing of Teflon collection filters was used to determine mass concentration of nPM. Inductively coupled plasma mass spectroscopy (ICP-MS) was used to analyze PM-bound metals and trace elements. GE Sievers 900 TOC analyzer (GE Sievers, Boulder, CO) was used to assay the water-soluble organic carbon. Further details regarding the procedures employed for the chemical analysis have been elaborated elsewhere [40].

### Immunofluorescence

Paraffin embedded samples were sliced into 5 μm-thick coronal sections from a portion of the brain located from 1 mm anterior to the bregma to 2 mm posterior to the bregma. These sections were selected for immunofluorescent staining. Sections were deparaffined and rehydrated with graded alcohol solutions ranging from 100–70%. Antigen retrieval was achieved by heating with Dako antigen retrieval solution, or 10mM sodium citrate. Sections were then blocked with 5% donkey serum and incubated overnight with primary antibody. Reactive microglia were identified by double staining with Iba-1 (anti-rabbit, 1:200, Wako) and either iNOS (anti-mouse, 1:200, BD Biosciences 610329) or arginase-1 (anti-goat, 1:250, Novas NBP1-36936). Antibodies used included degraded myelin basic protein (dMBP) (anti-rabbit, 1:500, EMD Millipore AB5864) for white matter, oligodendrocyte transcription factor 2 (Olig2) for oligodendrocytes (anti-goat, 1:200, R&D Systems AF2418), and SMI-312 neurofilament (anti-mouse, 1:500, BioLegend 837904) for axons. Sections were then incubated with appropriate Alexa Fluor secondary antibody (1:200, Thermo Fisher Scientific) and counter-stained with Hoechst 33342 to identify nuclei (1:5000, Life Technologies). Slides were mounted with Dako fluorescent mounting media and coverslipped.

The corpus callosum is the largest white matter commissure connecting the two cerebral hemispheres which plays a causal role in maintaining interhemispheric functional connectivity in humans [41]. Given its size and functional importance in humans, the corpus callosum was selected as the primary area of interest in these experiments. The medial corpus callosum was visualized with BZ-9000 fluorescent microscopy (Keyence, NJ) at 400x. For dMBP immunofluorescence, the optic tract and internal capsule were imaged in addition to the medial corpus callosum. Olig2 positive cells, Iba-1 positive cells, and cells that co-localized Iba-1 and either iNOS or arginase-1 (Arg) were counted and recorded. Mean fluorescent densities of dMBP and SMI-312 neurofilament were quantified. For each outcome measure, one section per animal was used, and values from the right and left medial corpus callosum were averaged. For assessment of dMBP immunofluorescent density, values from the right and left internal capsule, optic tract, and medial corpus callosum were averaged. Two independent, blinded observers performed the analysis, and scores were averaged to calculate one value per animal. Image analysis was performed using Image J software (NIH), and all protocols adhered to Image J user guide.

### Statistical analysis

All analyses were performed by investigators blinded to the exposure cohort. Tests for normality and homoscedasticity were performed. All data sets were normally distributed and had equal variance. Group means of filter and nPM were compared using two-tailed unpaired student’s t-tests. Pearson’s correlation coefficients were calculated to evaluate the relationships between microglia cell counts and white matter immunofluorescent density. The Grubb’s test (alpha = 0.05) was used to study outliers. No outliers were identified. Statistics were performed using GraphPad Prism software. Data are presented as mean ± standard deviation. Alpha of less than 0.05 is used to indicate statistical significance.

## Results

### nPM composition

nPM composition was analyzed to determine the exposure aerosol size and chemical composition associated with the present study’s results. During the 150 hours of exposure, the average mass concentration was 330 ± 25 μg/m^3^, and the average particle number concentration (PNC) was 1.6 (±0.3) * 10^5^ particles/cm^3^. Total organic carbon was the most predominant chemical species, accounting for 68% of total mass. The mass fractions of each of the trace elements and metals are listed in Table 1. The size distribution of the exposure aerosol is presented in Table 2. According to the table, the mode diameter is around 50 nm which is typical of particulate matter in the urban areas impacted by traffic emissions [42, 43].

**Table 1:** Mass fraction of the elements and metals (ng/μg PM) during exposures.

Species	Mean	Standard deviation	Species	Mean	Standard deviation
Na	36.88	0.46	Mg	10.34	0.02
Al	8.85	0.04	S	37.69	0.27
K	6.67	0.07	Ca	33.28	0.35
Ti	0.35	0.04	V	0.04	0.00
Cr	0.16	0.00	Mn	0.33	0.00
Fe	8.65	0.07	Ni	0.26	0.01
Cu	0.58	0.01	Zn	2.98	0.02
Ba	0.75	0.01	Pb	0.11	0.00

**Table 2:** Size distribution of the aerosol during exposures.

	Mean	Standard deviation
Mode (nm)	53.3	3.9
Median (nm)	55.2	0.6
Mean (nm)	68.0	0.7
Geometric mean (nm)	55.9	0.5
Geometric standard deviation	1.8	-

### Microglial response

**Fig 1:**
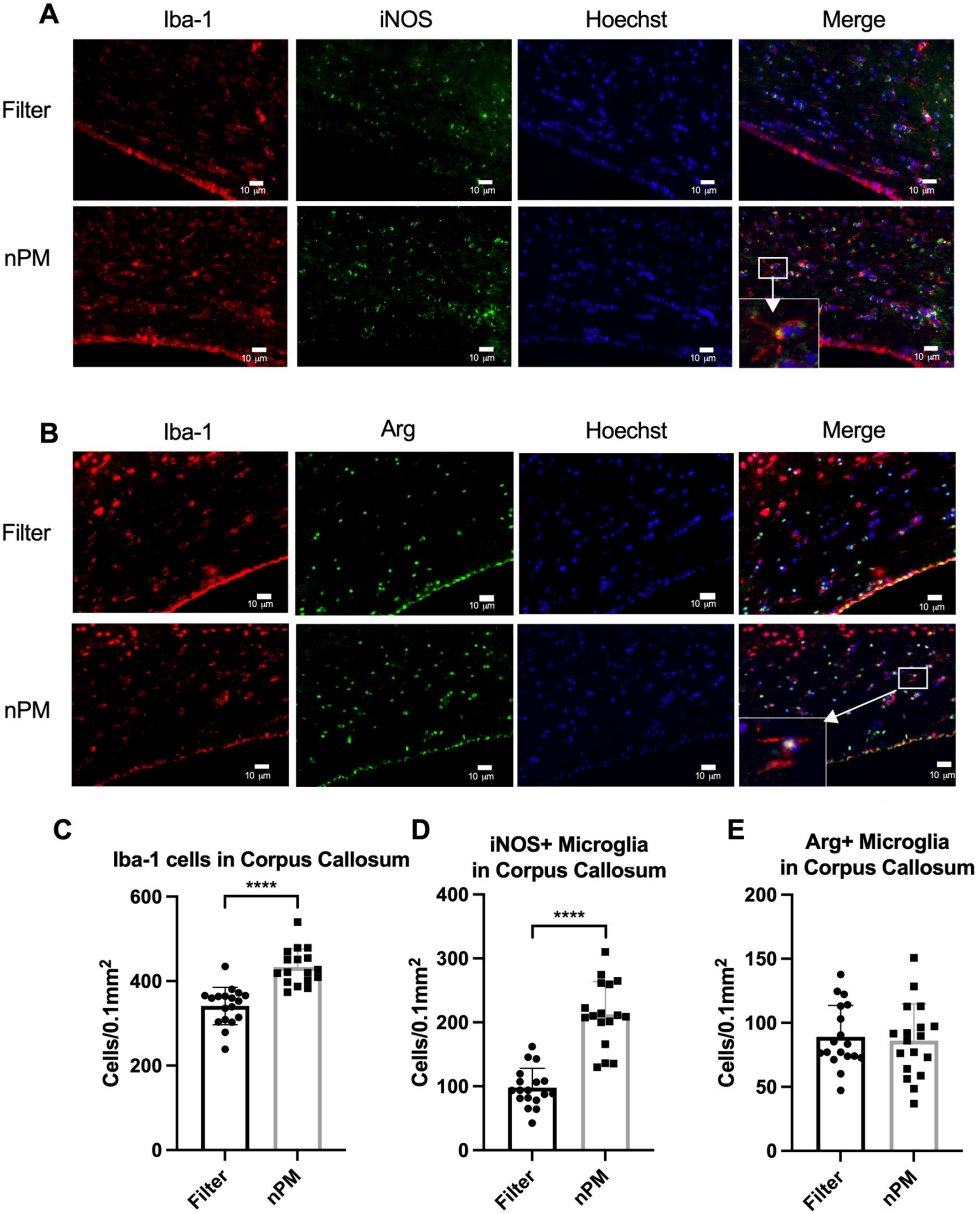
nPM exposure results in an inflammatory microglia response in the corpus callosum. (A) Iba-1+iNOS microglia in the corpus callosum of mice exposed to filtered air and nPM, with representative image of a positive cell at high magnification. (B) Iba-1+Arg microglia in the corpus callosum of mice exposed to filtered air and nPM, with representative image of a positive cell at high magnification. (C) Iba-1 positive microglia cell counts were significantly increased in the corpus callosum of mice exposed to nPM (n = 17) compared to filtered air (n = 18) (p<0.0001). (D) iNOS positive microglia cell counts were significantly increased in the corpus callosum of mice exposed to nPM (n = 17) compared to filtered air (n = 18) (p<0.0001). (E) Arginase-1 positive microglia cell counts were not significantly different in the corpus callosum of mice exposed to nPM (n = 18) compared to filtered air (n = 18) (p = NS). Data presented as mean ± standard deviation. Scale bars represent 10 μm. Error bars represent standard deviation. **** signifies p<0.0001.

Microglial reactivity following nPM exposure was assessed using histological analysis. A 1.3-fold increase in Iba-1 positive cells was demonstrated in the corpus callosum of mice exposed to nPM (433.13±42.92, n = 17) when compared to those exposed to filtered air (341.14±44.31, n = 18) (p<0.0001). iNOS-expressing microglia were increased by 2.2-fold in the corpus callosum of mice exposed to nPM (212.58±51.19, n = 17) when compared to filtered air (97.85±30.30, n = 18) (p<0.0001) (Fig 1). There was no significant difference in the number of arginase-expressing microglia in the corpus callosum of mice exposed to nPM and filtered air (nPM: 86.05±28.69, n = 18; filter: 89.13±24.54, n = 18; p = 0.73) (Fig 1).

### White matter injury and axonal degradation

nPM-mediated white matter and axonal injury were quantified in the corpus callosum. Immunohistochemistry was performed for the markers dMBP and Olig2 to assess for myelin and oligodendrocyte damage, respectively. Olig2 is a transcription factor expressed in all oligodendrocytes, including oligodendrocyte precursor cells (OPCs) and mature oligodendrocytes [44]. Histochemical analysis was performed on the pan-axonal neurofilament marker SMI-312 to assess for axonal injury.

**Fig 2:**
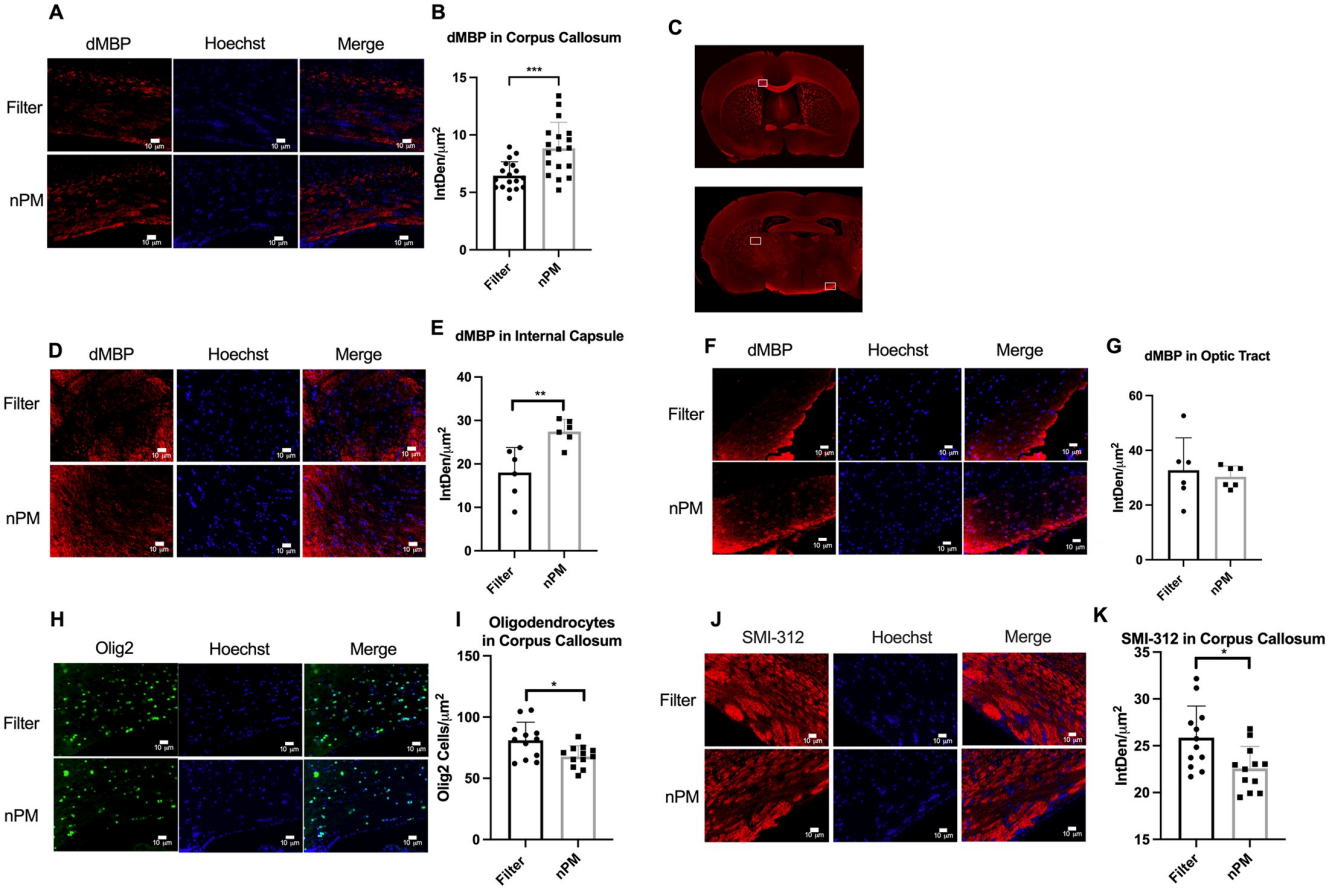
nPM exposure results in myelin injury and axonal damage in the corpus callosum. (A) dMBP in the corpus callosum of mice exposed to filtered air and nPM. Analysis was performed on both right/left sides. Right side location is marked in C. (B) dMBP immunofluorescent density was significantly increased in the corpus callosum of mice exposed to nPM (n = 18) compared to filtered air (n = 18) (p<0.001). (C) Representative images of regions analyzed (Top: Right side corpus callosum is marked. Bottom: Right side internal capsule and left side optic tracts are marked). Analysis was performed on both right and left sides. (D) dMBP in the internal capsule of mice exposed to filtered air and nPM. Analysis was performed on both right/left sides. Right side location is marked in C. (E) dMBP immunofluorescent density was significantly increased in the internal capsule of mice exposed to nPM (n = 6) compared to filtered air (n = 6) (p<0.01). (F) dMBP in the optic tract of mice exposed to filtered air and nPM. Analysis was performed on both right/left sides. Left side location is marked in C. (G) dMBP immunofluorescent density was not significantly different in the optic tract of mice exposed to nPM (n = 6) compared to filtered air (n = 6) (p = 0.65). (H) Oligodendrocytes in the corpus callosum of mice exposed to filtered air and nPM. (I) Oligodendrocyte cell counts were significantly decreased in the corpus callosum of mice exposed to nPM (n = 12) compared to filtered air (n = 12) (p<0.05). (J) SMI-312 in the corpus callosum of mice exposed to filtered air and nPM. Analysis was performed on both right/left sides. (K) SMI-312 immunofluorescent density was significantly decreased in the corpus callosum of mice exposed to nPM (n = 12) compared to filtered air (n = 12) (p<0.05). Data presented as mean ± standard deviation. Scale bars represent 10 μm. Error bars represent standard deviation. *signifies p<0.05, **signifies p<0.01, *** signifies p <0.001.

Mice exposed to nPM had significant white matter injury and axonal degradation in the corpus callosum. dMBP immunofluorescent density was increased by 1.4-fold in the corpus callosum of mice exposed to nPM (8.83±2.28, n = 18) compared to filtered air (6.45±1.22, n = 18) (p<0.001) (Fig 2). Oligodendrocyte cell counts were decreased by 1.16 fold in the corpus callosum of mice exposed to nPM (678.6±88.17, n = 12) compared to filtered air (810.6±147.7, n = 12) (p<0.05). Axonal neurofilament marker SMI-312 immunofluorescent density was decreased by 1.13 fold in the corpus callosum of mice exposed to nPM (22.57±2.37, n = 12) compared to filtered air (25.85±3.39, n = 12) (p<0.05)(Fig 2).

To provide a more comprehensive assessment of white matter injury, nPM-mediated white matter neurotoxicity was assessed in the internal capsule and optic tract with dMBP histochemical analysis. dMBP immunofluorescence density was increased by 1.5-fold in the internal capsule of mice exposed to nPM (27.5±2.82, n = 6) compared to filtered air (18.0±5.76, n = 6) (p<0.01). dMBP immunofluorescence density was not significantly increased in the optic tract of mice exposed to nPM (32.7±11.86, n = 6) compared to filtered air (30.3±3.97, n = 6) (p = 0.65) (Fig 2).

### Correlation of microglial reactivity and myelin injury

**Fig 3:**
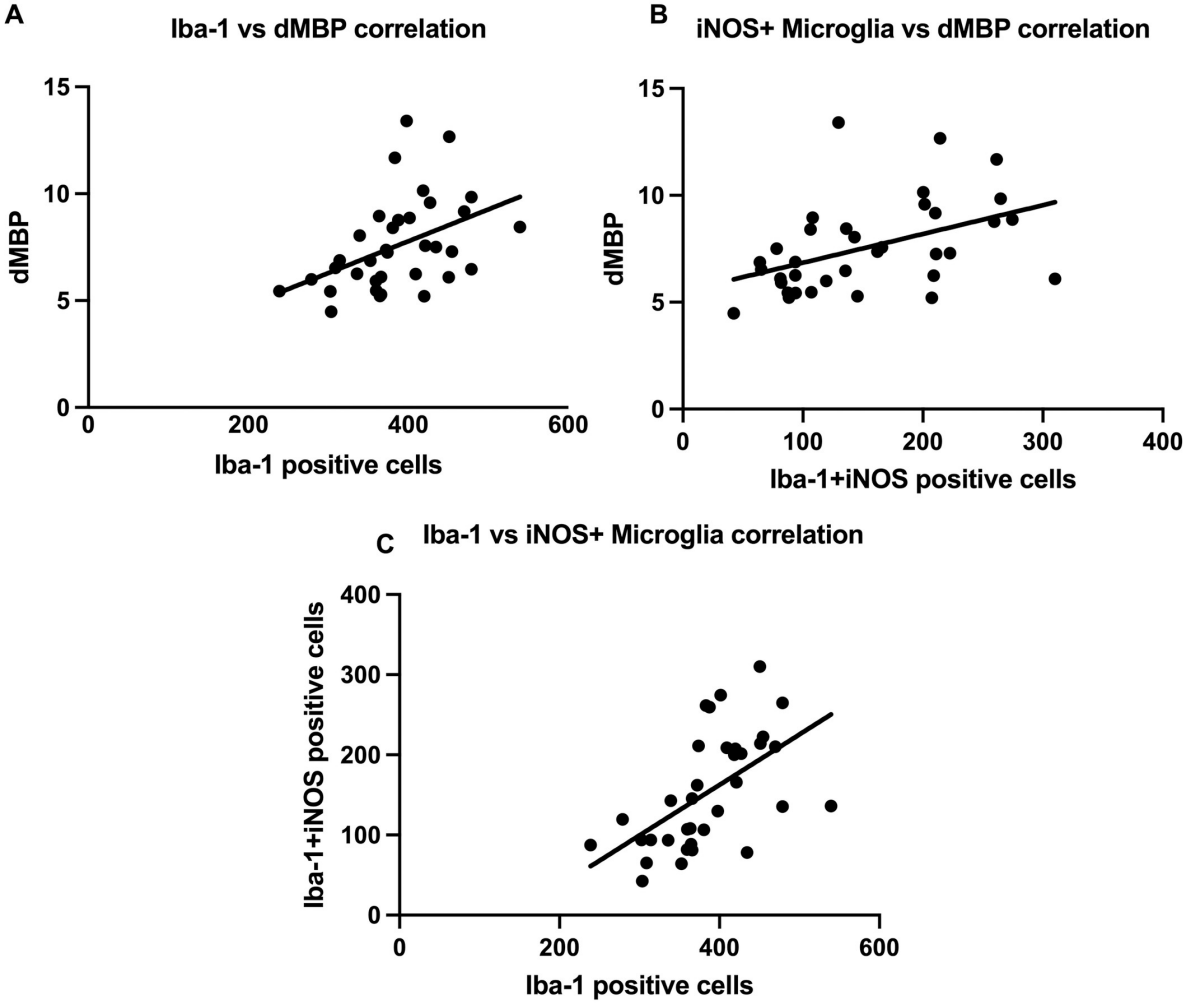
Microglial reactivity is correlated with myelin injury in the corpus callosum. (A) Iba-1 positive cell count was positively correlated with dMBP immunofluorescent density (r(33) = 0.438, p = 0.009). (B) iNOS-expressing microglia cell count was positively correlated with dMBP immunofluorescent density (r(33) = 0.445, p = 0.007). (C) Iba-1 positive cell count was positively correlated with iNOS-expressing microglia cell count (r(33) = 0.560, p<0.001).

The association between reactive microglia and myelin injury was investigated to determine if microglia may influence myelin injury. Iba-1 positive cell count in the corpus callosum was positively correlated with dMBP immunofluorescent density (r(33) = 0.438, p = 0.009)(Fig 3A). iNOS-expressing microglia cell count was positively correlated with dMBP immunofluorescent density (r(33) = 0.445, p = 0.007)(Fig 3B). Arginase-expressing microglia cell count was not correlated with dMBP immunofluorescent density. Iba-1 positive cell count was positively correlated with iNOS-expressing microglia cell count (r(33) = 0.560, p<0.001) (Fig 3C).

## Discussion

Our data demonstrates that ten weeks of nPM exposure causes white matter injury, axonal damage, and a reactive microglial response in the corpus callosum of adult male mice. Increases in total microglia counts were noted in the nPM-exposed cohort and higher microglial cell counts were associated with greater burden of myelin injury in individual mice. Higher cell counts of pro-inflammatory iNOS-expressing microglia were observed in the corpus callosum of nPM-exposed mice while no differences in anti-inflammatory/ homeostatic arginase-expressing microglia were detected.

### nPM exposure may induce white matter injury

Human studies suggest that white matter may be particularly susceptible to air pollution-induced neurotoxicity (specifically PM_2.5_) across age ranges. Prenatal exposure to elevated levels of PM_2.5_ is associated with decreased corpus callosum volumes in children [45]. Both children and dogs exposed to high levels of ambient air pollution in Mexico City demonstrated pre-frontal white matter pathology (and cognitive deficits in the children) when compared to those who resided in areas with lower levels of pollution [3, 21]. In the Women’s Health Initiative Memory Study (WHIMS), increased estimated exposure to fine particulate matter (<2.5 μm) was associated with decreased white matter volumes on MRI [20, 46]. Region of interest analysis specifically demonstrated white matter volumetric changes in the corpus callosum and frontal/ temporal lobes [20].

Experimental studies examining the relationship between toxic exposures and white matter pathology have noted similar associations [18, 19, 47]. Further, these studies suggest that white matter damage may be mediated by neuroinflammation [3, 14, 19]. Young female mice exposed to 150 hours of traffic-related air pollution (TRAP, nanoparticulate matter <0.2 μm diameter) had increased Iba-1 integrated density, with associated neurite atrophy, increased TNFα mRNA, and decreased myelin basic protein in the hippocampus [19]. Pathological changes in myelin sheaths have been observed in the cortex of rats exposed to intratracheal PM_2.5_ (<2.5 μm) with greater myelin disorganization after longer exposure periods [18]. Apoptotic and reactive microglia and astrocytic cells were seen in the white matter of dogs in a highly polluted area of Mexico City [47].

Particulate matter- induced white matter injury is likely dependent on exposure duration. Our study found that adult male mice exposed to 10 weeks (150 hours) of nPM demonstrated significant myelin and oligodendrocyte damage in the corpus callosum, as evidenced by increased dMBP immunofluorescent density and decreased oligodendrocyte cell counts. In the same experimental model, 45 hours of nPM exposure resulted in inflammatory and oxidative stress responses in the olfactory bulb and olfactory epithelium, while other brain regions, such as the cortex and cerebellum, were affected later in the time course [15]. Taken together, this data suggests that an extended exposure may be required to cause injury in post-olfactory regions of the brain, such as the corpus callosum. Further, the present study suggests that nPM-mediated white matter injury may vary throughout cerebral white matter tracts. We demonstrated that chronic nPM exposure induced myelin injury in the corpus callosum and internal capsule of adult mice. nPM exposure did not induce white matter injury in the optic tract. However, the sample size was small (n = 6/group), which may have influenced results.

### nPM exposure may produce neurite dysfunction

Experimental and in-vitro studies have suggested that nPM exposure may result in neurite dysfunction. nPM exposure in female EFAD and C57BL/6 mice resulted in selective hippocampal CA1 neurite atrophy [8, 19]. Microglia-derived TNF-α may mediate this neurite dysfunction. nPM-exposed microglial cultures exhibited an increase in microglia-derived TNF-α, which subsequently inhibited neurite outgrowth [48]. Data from the present study suggests that nPM exposure results in myelin injury and detachment of myelin from axons in the corpus callosum. Subsequently, this separation may contribute to axonal degradation as demonstrated by decreased neurofilament SMI-312 immunofluorescent density.

### Microglial response to nPM exposure

It is well recognized that air pollution exposure induces inflammation and oxidative stress in the central nervous system [3, 13–17]. Microglia play a critical role in this response, and may directly contribute to white matter injury [14, 15, 19]. Previous studies demonstrated both increased numbers of microglia and reactive morphologic alterations in response to particulate matter exposures [14, 15]. We have demonstrated that this microglial response can occur in the absence of astrocytic changes [14]. Based on this data, we focused our experiments on the microglial response to nPM in the current study and examined proinflammatory and homeostatic phenotypes by double staining.

### Microglial response to injury

In response to injury, microglia undergo rapid alterations in gene expression, function, and morphology [25, 26]. Prior studies have characterized microglia into either pro-inflammatory, or anti-inflammatory phenotypes [28]. A biphasic microglial response has been described in experimental models of central nervous system injury. Miron et al. injected lysolecithin into the corpus callosum of mice to induce focal demyelination, and characterized the microglial response at post-lesion day 3, 10, and 21 [29]. An initial increase in the inflammatory iNOS-expressing phenotype was observed. This was followed by a transition to the anti-inflammatory arginase-expressing phenotype, concordant with re-myelination at day 10 [29]. A divergent microglial response was similarly noted in mice that had undergone spinal cord injury [49]. Gene expression studies and histochemical analyses demonstrated that inflammatory iNOS-expressing microglia rapidly increased post-injury and persisted for up to 1 month. Anti-inflammatory microglial gene expression changes, however, were transient, and returned to pre-injury levels within one week [49]. Increasing evidence suggests that microglia may polarize into inflammatory and anti-inflammatory phenotypes after ischemic stroke. This polarization may contribute to infarct evolution and healing [26]. In an experimental murine stroke model, microglia demonstrated an anti-inflammatory arginase-expressing phenotype early in the post-ischemic period followed by an inflammatory iNOS-expressing phenotype [37].

The polarization to an inflammatory subtype may result from neuronal death, and subsequent release of toxic factors, which further contribute to the progression of ischemic damage [37]. Although this microglial nomenclature is limited to two phenotypes (inflammatory/anti-inflammatory), it broadly describes a range of cellular response. Research continues to further characterize nuances of the microglia response and recognizes that there may be multiple sub-types of surveillance, reactive, pro-inflammatory and anti-inflammatory microglia [28].

In the studies discussed above, the neurotoxic injury was acute. However, nPM exposure was continuous and persisted across ten weeks in our experimental paradigm. Microglial reactivity was analyzed by histochemical staining at the end of the study period. Increases in total and pro-inflammatory iNOS-expressing microglia were noted in the corpus callosum of nPM-exposed mice. There was no difference in anti-inflammatory arginase-expressing microglia between groups. This is likely due to the sustained nature of the exposure, which does not allow for a distinct recovery period.

### iNOS expression in microglia

Reactive microglia release neurotoxic substrates that contribute to inflammation and oxidative stress [28, 48, 49]. Studies suggest that iNOS expression is important in the microglial inflammatory response to nPM exposure [15]. We therefore selected iNOS as a marker of pro-inflammatory microglia in our study. iNOS produces large quantities of nitric oxide (NO), which is neurotoxic in excess [50]. Unlike other forms of nitric oxide synthase (NOS), such as endothelial NOS (eNOS), iNOS is not constitutively expressed [50, 51]. In response to injury, microglia can upregulate iNOS expression. NF-kB and STAT1 are transcription factors that are implicated in the microglial inflammatory response and can induce iNOS expression [52, 53]. In turn, iNOS production may contribute to the synthesis of inflammatory factors, including IL-6, TNFα, and CD14 [51, 54, 55].

The iNOS pathway in microglia has been implicated as a potential mechanism by which inflammatory neurodegeneration occurs [52, 56]. Kigerl et al. demonstrated that conditioned media from inflammatory iNOS-expressing microglia, but not anti-inflammatory arginase-expressing microglia, was toxic to neurons due to release of oxidative factors [49]. In our study, we found that increasing numbers of microglia, and specifically iNOS-expressing microglia, were associated with the extent of corpus callosum myelin injury.

### Arginase-1 expression in microglia

We selected arginase as a marker of anti-inflammatory microglia with potential neuroprotective capacity [30, 31, 54]. Arginase-1 is an enzyme that converts arginine into ornithine and urea. The resulting polyamines are necessary for tissue remodeling and cell proliferation [54, 57]. The overexpression of arginase-1 in microglia can decrease iNOS and NO expression, thereby reducing iNOS-mediated neuroinflammation [54, 57]. Cherry et al. demonstrated that arginase-1 positive microglia were involved with Aß plaque clearance during sustained neuroinflammation in a murine model [31]. Further, neuronal survival was associated with the number of arginase-1 positive microglia in a murine model of ischemic stroke [30].

### Limitations

There are several potential limitations in this study. Our study was conducted on exclusively male mice. Estrogen may influence microglial polarization, as well as protect against white matter injury [58–62]. In in-vitro studies, BV2 cells and primary cerebral cortex microglia adopted an inflammatory phenotype in the setting of hypoxia. Simultaneous administration of estrogen partially prevented this shift to an inflammatory phenotype [60–62]. The present study was unable to investigate the sex differences of nPM exposure due to sample size limitations. The pro-inflammatory/ anti-inflammatory classification may be oversimplified. These subtypes represent the extremes of a spectrum of intermediate microglia phenotypes. However, the pro-inflammatory/ anti-inflammatory dichotomy remains useful to understand the broad response of microglia throughout the stages of central nervous system injury [28]. All endpoints were assessed at the same time point. While an association between reactive microglia and white matter injury is demonstrated, we cannot describe the time-course of events and determine the causes of white matter injury. It is possible that nPM-mediated demyelination may induce an inflammatory microglial response. This microglial response may then induce neuroinflammation and further contribute to white matter neurotoxicity.

### Conclusion

Our results indicate that subacute exposure to nanoscale particulate matter can result in white matter injury in adult male mice. Microglial response and polarization towards the pro-inflammatory, iNOS-expressing phenotype may play an important role in this process. These findings suggest that reactive microglia may be relevant to interactions between air pollution and neurocognitive disease.

## Supporting information

S1 Dataset Iba-1, iNOS positive microglia, Arginase positive microglia, dMBP, Olig2, and SMI312 dataset. (XLSX)

S2 Dataset Correlation dataset. (XLSX)
